# Synthesis of Polyheterocyclic Dimers Containing Restricted and Constrained Peptidomimetics via IMCR-Based Domino/Double CuAAC Click Strategy

**DOI:** 10.3390/molecules25225246

**Published:** 2020-11-11

**Authors:** Shrikant G. Pharande, Manuel A. Rentería-Gómez, Rocío Gámez-Montaño

**Affiliations:** Departamento de Química, División de Ciencias Naturales y Exactas, Universidad de Guanajuato, Noria Alta S/N, Col. Noria Alta, 36050 Guanajuato, Mexico; shrikant.pharande@gmail.com (S.G.P.); vmx_rntclone@hotmail.com (M.A.R.-G.)

**Keywords:** isocyanide-based multicomponent reactions, IMCR-based domino and tandem process, CuAAC click, polyheterocyclic dimers, restricted and constrained peptidomimetics

## Abstract

A novel strategy via the triple process (multicomponent reactions (MCR)-domino)/tandem was developed for the synthesis of restricted and constrained bis-1,2,3-triazole-linked pyrrolo[3,4-*b*]pyridine peptidomimetics dimers in overall yields of 20–55%. This strategy allows the construction of six heterocycles in two stages of the reaction.

## 1. Introduction

The design of peptidomimetics has emerged as an important tool for medicinal chemists to address problems associated with natural peptides. In particular, the incorporation of cyclic scaffolds into constrained peptidomimetics is of high interest, as they decrease the flexibility of the peptide, reducing the number of conformations, thus enhancing their affinity and bioavailability for a certain receptor [1,2].

The restricted and constrained peptidomimetics play a central role in drug discovery and in the design of novel molecules with potential application in biological chemistry and are of particular interest in both academic and industry fields. In this context 1,4-disubstituted 1*H*-1,2,3-triazoles, which display structural and electronic similarities with the *trans*-amide bond, often enhance the biological activity of the parent molecule by increasing the metabolic stability and hydrogen-bonding ability. Furthermore, they are flat bivalent molecules, mimicking the restricted conformational constraints of double bonds in alkyl chains and can be used as a replacement of a variety of other five-membered nitrogen-containing heterocycles [3,4].

**Figure 1:**
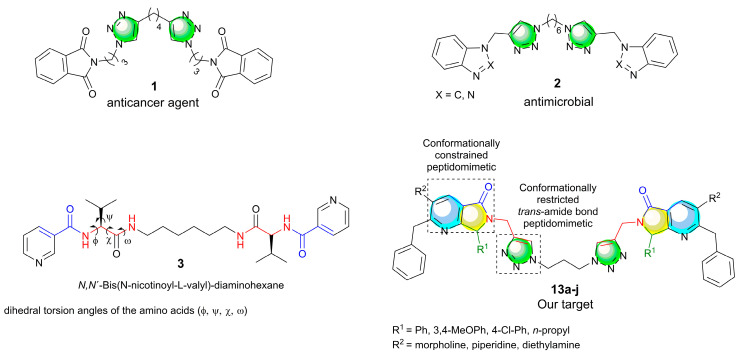
Bioactive, 1,2,3-triazole-linked dimers **1** and **2**, bis-(*N*-nicotinoyl-L-valyl, bis-derivatives of L-valine **3** and our target compound **13**.

Examples of bioactive triazole-linked dimeric heterocycles include anticancer agent **1** [5,6,7,8], antimicrobial **2** [9,10,11,12], as well as antioxidants [10] and antipsychotic agents [13] (Figure 1). It is important to note that these compounds have an aliphatic chain spacer between the 1,2,3-triazole rings, probably for lipophilic control, and a heterocyclic component linked to the triazole ring. Other applications are in coordination chemistry, biochemistry, and also in supramolecular chemistry [14,15].

The pyrrolo[3,4-*b*]pyridin-5-one is an important fragment for building conformationally constrained peptidomimetics. Compounds incorporating this fused heterocycle exhibit a wide range of biological activities including anti-diabetic agents [16], anticancer, analgesic, and therapeutic agents for central nervous system-related diseases such as Alzheimer’s, epilepsy, and schizophrenia [17,18,19,20]. Furthermore, Wager et al. reported the synthesis of their analogs with brain-selective radioligand properties [21].

On the other hand, nicotinic and alkyl fragments containing bis-derivatives of L-Valine **3** showed potent neuropharmacological activities [22,23,24,25,26,27]. In this context, the compounds **13** synthesized here can be a rigid analogue of **3** incorporating a conformationally constrained fragment (pyrrolo[3,4-*b*]pyridin-5-ones) and *trans*-amide bond peptidomimetics (1,4-disubstituted 1*H*-1,2,3-triazoles) (Figure 1).

Multicomponent reactions (MCRs) have proven to be an efficient approach in organic synthesis. Particularly, the isocyanide-based multicomponent reactions (IMCRs), such as the Ugi and Passerini reactions, are the most relevant for constructing peptidomimetics since they give access to linear peptides and depsipeptide-like structures. The post-MCR transformation strategy toward the synthesis of privileged heterocyclic peptidomimetics (PHPs) is well documented [28]. The use of orthogonal and bifunctional inputs in MCRs plays a central role by allowing a variety of transformations on the intermediates generated and thus increases the molecular complexity [29]. Among the all MCR strategies known to access restricted and/or constrained PHPs, the ones that involve MCRs coupled with other one-pot processes in consecutive or domino manner are the most efficient, versatile, robust, and ecofriendly. In this context, the strategies involving domino processes are particularly desirable, because the molecular complexity is significatively increased and the secondary products are reduced. Zhu et al. are the pioneers of the post-MCRs transformation-based domino strategy [30,31].

**Scheme 1:**
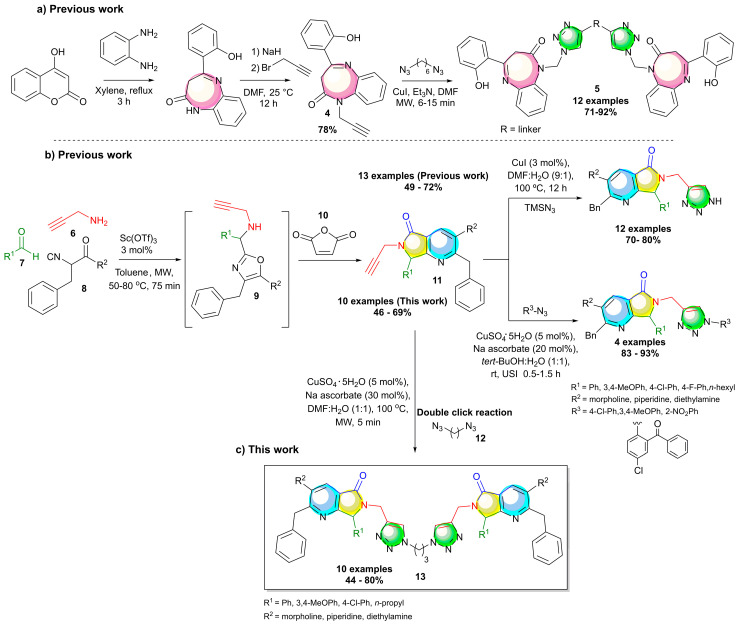
(**a**) Previous work for the synthesis of dimer of 1,5-benzodiazepine-1,2,3-triazole; (**b**) synthesis of PHPs **11** and pyrrolo[3,4-*b*]pyridin-5-ones linked to 1,2,3-triazole; and (**c**) their complex analogs dimer **13** via the sequence: isocyanide-based multicomponent reactions (IMCR)/aza Diels–Alder-based domino process/double CuAAC click process.

Our ongoing research program focuses on the design of new or novel, rapid, convergent, ecofriendly, and efficient post-IMCR/transformation strategies in consecutive [32,33,34,35,36,37,38] or domino manner [39,40,41] toward the synthesis of novel molecules containing conformationally restricted and/or constrained peptidomimetics. Recently, we reported the first ultrasound-assisted green one-pot synthesis of molecules containing privileged restricted peptidomimetics via this strategy: post-IMCR transformation/ Copper(I)-catalyzed azide-alkyne cycloaddition (CuAAC) click reaction employing a green alternative energy source [42]. In addition, the synthesis of molecules containing restricted and constrained PHPs via IMCR, followed by a domino process and subsequent CuAAC to incorporate 1,2,3-triazoles moiety (Scheme 1b) [32]. Surprisingly, the synthesis of PHPs via the post-MCR transformation click strategy has rarely been reported [43,44].

To our knowledge, the strategy developed to synthesize dimers via a repetitive IMCR employing a bifunctional starting material is well documented [45,46,47,48,49]. The coupling of MCR with other domino processes (MCR-based domino) is undeniably the best strategy to increase their synthetic potential and to generate molecular complexity. However, their application in the design and development of more efficient and ecofriendly strategies toward the synthesis of complex molecules such as polyheterocyclic dimers is practically unexplored. To date, only three reports are available. [50,51,52].

Concerning the syntheses of dimers of 1,2,3-triazole, only multistep syntheses have been documented. In 2019, Msaddek and co-workers reported the synthesis to the dimers of 1,5-benzodiazepine-1,2,3-triazole (Scheme 1a) [10].

Encouraged by the fact that the post-MCR transformation strategy coupled to a double CuAAC click reaction for the synthesis of polyheterocyclic dimers has not been reported, we herein report a novel strategy toward the synthesis of new PHP dimers via a triple process: (MCR-domino)/tandem involving an IMCR coupled to a domino process followed by tandem process involving the Ugi 3-CR coupled to the aza Diels–Alder/*N*-acylation/decarboxylation/dehydration/aromatization) domino process followed by the double CuAAC click process. The developed strategy allowed us to synthesize polyheterocyclic dimers containing both restricted and constrained PHPs (Scheme 1c).

**Scheme 2:**
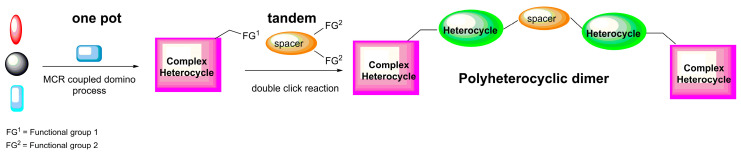
General synthetic strategy via a novel triple process: multicomponent reactions (MCR) coupled to a domino process followed by a tandem process toward polyheterocyclic dimers containing restricted and constrained peptidomimetics.

The main advantage of the strategy developed here is the coupling of three of the best efficient synthetic tools, improving the synthetic potential of each these processes, which allowed increased diversity and molecular complexity. The complex alkynes, playing a central role as precursors for click reactions, were synthesized from alicyclic starting reagents via the IMCR/aza Diels–Alder-based domino process. Then, the complex alkynes were subjected to the double CuAAC click reaction toward dimers containing 1,2,3-triazole-linked to other PHPs, increasing their potential in both synthetic and medicinal chemistry fields (Scheme 2). It is worth highlighting that the synthesized dimers contain restricted and constrained PHPs, which is an amazing result from the synthetic point of view considering that six heterocycles were constructed in only two reaction stages.

## 2. Results and Discussion

In this work, we report the two-step synthesis of dimer compound **13**, which contains three different heterocycles: pyridine, pyrrolidin-2-one, and 1,4-disubstituted 1*H*-1,2,3-triazole (Scheme 1). In the first step, the synthesis of **11** occurs via the Ugi-3CR followed by the aza Diels–Alder/*N*-acylation/decarboxylation/dehydratation/aromatization domino process to give a complex terminal alkyne functionalized at the α-position with a fused heterocycle.

**Scheme 3:**
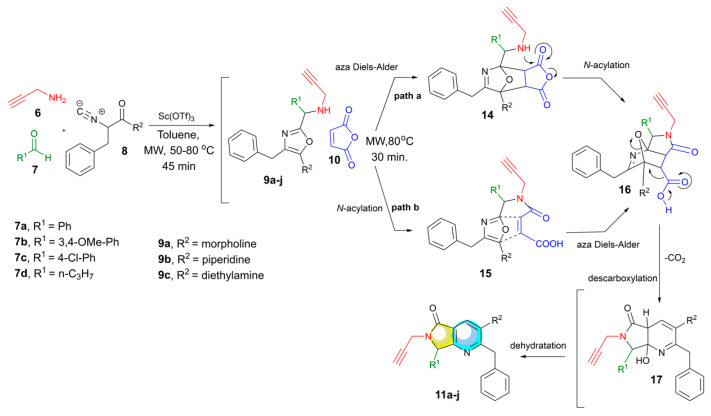
The plausible reaction mechanism for the formation of pyrrolo[3,4-*b*]pyridin-5-ones.

The plausible reaction mechanism for the formation of pyrrolo[3,4-*b*]pyridin-5-ones is shown in Scheme 3 and is supported by computational calculations performed using Density Functional Theory (DFT) methods [53]. The use of Lewis acids to activate imines has proven useful in Ugi-3CR with α-isocyano acetamides as reported by Zhu and co-workers [54], as the resulting iminium ions are more reactive than imines in the Ugi-3CR. Thus, after some reactions and with previously optimized reaction conditions [32,33,34], propargyl amine **6** was combined with aldehyde **7** to give the imine. Heating this imine at 50 °C for 30 min in microwave (MW) with 3 mol% Sc(OTf)_3_ resulted in iminium ion, which was then reacted with the α-isocyanoacetamide **8** at 80 °C for 15 min to give key 5-aminooxazole **9** via chain-ring tautomerization. This was followed by a domino process between **9** and maleic anhydride (**10**) via an aza-Diels–Alder/*N*-acylation/decarboxylation/dehydratation/aromatization sequence in the same pot at 80 °C for 30 min. It is highlighting that Sc(OTf)_3_ is an efficient catalyst performing a double role in the IMCR and in the aza-Diels–Alder cycloaddition process [55]. Complex alkynes functionalized with pyrrolo[3,4-*b*]pyridin-5-ones **11** were obtained in moderate-to-good yields (46–69%). The lowest yield of all the synthesized analogues was obtained with *p*-chlorobenzaldehyde (R^1^ = p-ClPh) and R^2^ = dimethylamino (Table 1).

**Scheme 4:**
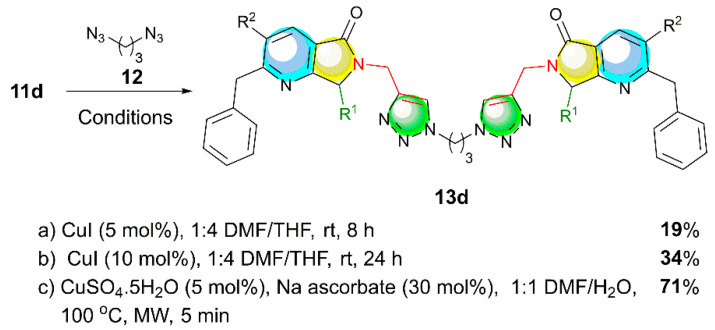
Optimization of the azide–alkyne cycloaddition.

Encouraged by the efficiency of the domino processes, we set out to explore the conditions that would enable its coupling with a tandem process via a double CuAAC click reaction using purified products **11** and 1,3-diazidopropane (**12**) (Scheme 4). When **11d** was reacted with 1,3-diazido propane **12** in the presence of CuI (5 mol%) at room temperature in 1:4 DMF/THF for 8 h, the desired product **13d** was formed in a low yield of 19%. Upon increasing the catalyst loading to 10 mol% and the reaction time to 24 h, the yield was improved to 34%. Fortunately, when the reaction was carried out at 100 °C in MW for 5 min, in 1:1 DMF/H_2_O with CuSO_4_•5H_2_O and sodium ascorbate, the product yield increased to 71%.

Thus, with the optimized conditions, the desired propane-linked bis-triazolyl-pyrrolo[3,4-*b*]pyridin-5-ones **13a**–**j** were prepared in 44–80% yields from pyrrolo[3,4-*b*]pyridin-5-ones **11a**–**j** (Table 1). Contrary to our previous report, MW irradiation at 100 °C allowed generation of the products in short reaction times of 5 min [32]. All the synthesized products were characterized by ^1^H and ^13^C-NMR and HRMS (compounds **13a**–**j** are shown in the Supplementary Material). It is known that the regiocontrol of azide–alkyne cycloadditions strongly depends on the nature of the catalysts and the reagents employed [56,57,58,59,60]. Copper(I) salts afford exclusively 1,4-adducts, while ruthenium cyclopentadienyl complexes promote 1,5-adduct formation [61,62]. In order to suggest the regioselectivity in triazole formation, we have compared the spectral data of our compound with literature values. A comparison of our ^1^H-NMR spectra with literature values confirmed the production of 1,4-regioisomers in each case

## 3. Materials and Methods

### 3.1. Materials

^1^H and ^13^C-NMR spectra were acquired on Bruker avance III (500 MHz) spectrometers. The solvent used was deuterated chloroform (CDCl_3_). Chemical shifts are reported in parts per million (δ/ppm). The internal reference for ^1^H-NMR spectra is tetramethylsilane (TMS) at 0.0 ppm. The internal reference for ^13^C-NMR spectra is CDCl_3_ at 77.0 ppm. Coupling constants (*J*) are reported in Hertz (Hz). Multiplicities of the signals are reported using the standard abbreviations: singlet (s), doublet (d), triplet (t), quartet (q), and multiplet (m). Nuclear magnetic resonance (NMR) spectra were analyzed using MestreNova software version 10.0.1-14719. Mass spectrometry (MS) spectra were acquired on a Bruker Daltonics Maxis Impact ESI-qTOF MS spectrometer. High-resolution mass spectrometry (HRMS) samples were ionized in electrospray ionization (ESI) mode and recorded via the time-of-flight (TOF) method. Reaction progress was monitored by thin-layer chromatography (TLC) on precoated silica gel Kieselgel 60 F254 plates, and the spots were visualized under UV light (254 or 365 nm). Flash column chromatography was performed using silica gel (230–400 mesh) and mixtures of hexanes with EtOAc in different proportions (*v*/*v*) or DCM with methanol (9:1 *v*/*v*) as the mobile phase. Melting points were determined on a Fisher–Johns apparatus and were uncorrected. All starting materials were purchased from Sigma-Aldrich and were used without further purification. Chemical names and drawings were obtained using the ChemBioDraw Ultra 13.0.2.3020 software package. The solvents were distilled and dried according to standard procedures.

### 3.2. Synthetic Procedures

#### 3.2.1. General procedure for the synthesis and characterization of the 6-Propargyl-pyrrolo[3,4-b]pyridin-5-ones **11a–j **(GP-1)

The propargylamine **6** (1.0 equiv.) and the corresponding aldehyde **7a**–**d** (1.0 equiv.) were placed in a 10 mL sealed CEM Discover^TM^ microwave reaction tube and diluted in 1.0 mL toluene. Then, the mixture was irradiated (MW, 60 W 50 °C) for 15 min, and Sc(OTf)_3_ (3% mol) was added. The mixture was irradiated (MW, 60 W, 50 °C) for 15 min, and the corresponding isocyanide **8a**–**c** was added (1.2 equiv.) was added. The mixture was irradiated (MW, 150 W, 80 °C), but this time for 30 min, and maleic anhydride (**10**) (1.4 equiv.) was added. Finally, this reaction mixture was irradiated (MW, 150 W, 80 °C) for 30 min. Then, the solvent was removed to dryness under vacuum. The crude product was purified by flash chromatography to afford the corresponding pyrrolo[3,4-*b*]pyridin-5-ones **11a**–**j**. For the characterizeation, see Gámez-Montaño*, *Front. Chem*. **2019**, 7:546.

#### 3.2.2. General procedure for the synthesis and characterization of the Propane-linked *bis*-Triazolyl-pyrrolo[3,4-*b*]pyridin-5-ones **13a**–**j** (GP-2)

The corresponding pyrrolo[3,4-*b*]pyridin-5-one **11a**–**j** (1.0 equiv.) and 1,3-diazido propane (0.5 equiv.) were placed in a 10 mL sealed CEM Discover^TM^ microwave reaction tube and diluted in 1.0 mL DMF:H_2_O (1:1), CuSO_4_•5H_2_O (5 mol%) and sodium ascorbate (30 mol%) were added. The mixture was irradiated (MW, 150 W, 100 °C) for 5 min. Next, the reaction mixture was diluted in water and extracted with ethyl acetate. The organic layer was dried over anhydrous Na_2_SO_4_ and concentrated under vacuum to afford the crude product. The residue was purified by flash chromatography using MeOH–dichloromethane (10% MeOH in dichloromethane) as eluent to give propane-linked bis-triazolyl-pyrrolo[3,4-*b*]pyridin-5-one **13a**–**j**.

*6,6′-((Propane-1,3-diylbis(1H-1,2,3-triazole-1,4-diyl))bis(methylene))bis(2-benzyl-3-morpholino-7-phenyl-6,7-dihydro-5H-pyrrolo[3,4-b]pyridin-5-one)* (**13a**): According to GP-2, **11a** (50 mg, 0.0011 μmol), 1,3-diazido propane (**12**) (7.4 mg, 0.059 μmol), CuSO_4_•5H_2_O (2.9 mg), and Na ascorbate (7.02 mg, 0.006 μmol) were reacted together in 1.0 mL DMF:H_2_O (1:1) in MW to afford the propane-linked bis-triazolyl-pyrrolo[3,4-b]pyridin-5-ones **13a** (58 mg, 50%) as yellow solid; m.p. 197–198 °C; DCM-MeOH = 9/1 v/v; ^1^H-NMR (400 MHz; CDCl_3_; 25 °C; TMS): δ 7.86 (s, 2H), 7.61 (s, 2H), 7.44–7.32 (m, 6H, Ar-H), 7.31–7.20 (m, 5H, Ar-H), 7.18–7.10 (m, 9H, Ar-H), 5.63 (s, 2H), 5.20 (d, 2H, J = 15.4 Hz), 4.41–4.23 (m, 6H), 4.22–4.09 (m, 4H), 3.89–3.63 (m, 8H), 2.92–2.68 (m, 8H), 2.55–2.41 (m, 2H); ^13^C-NMR (100 MHz, CDCl_3_) δ 167.1, 162.3, 160.6, 147.8, 143.8, 139.2, 135.2, 129.0, 128.8, 128.3, 128.2, 126.2, 123.8, 123.4, 67.1, 65.4, 53.0, 46.8, 40.0, 35.4, 30.4; HRMS (ESI+): m/z calcd. for C_57_H_56_N_12_O_4_^+^: 973.4620, found: 973.4599.

*6,6′-((Propane-1,3-diylbis(1H-1,2,3-triazole-1,4-diyl))bis(methylene))bis(2-benzyl-7-(3,4-dimethoxyphenyl)-3-morpholino-6,7-dihydro-5H-pyrrolo[3,4-b]pyridin-5-one)* (**13b**): According to GP-2, **11b** (50 mg, 0.0010 μmol) 1,3-diazido propane (**12**) (6.5 mg, 0.051 μmol), CuSO_4_•5H_2_O (2.5 mg), and Na ascorbate (6.15 mg, 0.006 μmol) were reacted together in 1.0 mL DMF:H_2_O (1:1) in MW to afford the propane-linked bis-triazolyl-pyrrolo[3,4-b]pyridin-5-ones (**13b**) (71 mg, 63%) as orange solid; m.p. 222–224 °C; DCM-MeOH = 9/1 v/v; ^1^H-NMR (500 MHz, CDCl_3_) δ 7.81 (s, 2H), 7.55 (s, 2H), 7.12–7.02 (m, 20H, Ar-H), 6.81 (s, 2H), 6.59 (s, 2H), 5.54 (s, 2H), 5.10 (d, 2H, J = 5.11 Hz), 4.27–4.08 (m, 6H), 3.81 (s, 6H), 2.90–2.70 (m, 8H), 2.55–2.35 (m, 2H); ^13^C-NMR (126 MHz, CDCl_3_) δ 167.0, 162.3, 160.6, 149.4, 147.8, 143.8, 139.2, 128.8, 128.2, 127.3, 126.2, 123.8, 123.4, 121.2, 111.4, 110.8, 67.1, 65.2, 56.0, 55.9, 53.0, 46.8, 40.1, 35.2, 29.7; HRMS (ESI+): m/z calcd. for C_61_H_64_N_12_O_8_^+^: 1093.5042, found: 1093.5010.

*6,6′-((Propane-1,3-diylbis(1H-1,2,3-triazole-1,4-diyl))bis(methylene))bis(2-benzyl-7-(4-chlorophenyl)-3-morpholino-6,7-dihydro-5H-pyrrolo[3,4-b]pyridin-5-one)* (**13c**): According to GP-2, **11c** (50 mg, 0.00109 μmol), 1,3-diazido propane **12** (6.9 mg, 0.054 μmol), CuSO_4_•5H_2_O (2.7 mg), and Na ascorbate (6.5 mg, 0.0032 μmol) were reacted together in 1.0 mL DMF:H_2_O (1:1) in MW to afford the propane-linked bis-triazolyl-pyrrolo[3,4-b]pyridin-5-ones (**13c**) (90 mg, 79%) as beige solid; m.p. 201–203 °C; DCM-MeOH = 9/1 v/v; ^1^H-NMR (500 MHz, CDCl_3_) δ 7.78 (s, 2H), 7.61 (s, 2H), 7.34 (d, J = 8.4 Hz, 4H), 7.20 (d, J = 8.3 Hz, 4H), 7.18–7.11 (m, 10H), 5.61 (s, 2H), 5.19 (d, J = 15.4 Hz, 2H), 4.34–4.26 (m, 6H), 4.34–4.26 (m, 6H), 3.84–3.76 (m, 6H), 2.85–2.76 (m, 8H), 2.55–2.41 (m, 2H); ^13^C-NMR (126 MHz, CDCl_3_) δ 167.2, 162.6, 160.2, 148.1, 143.7, 139.2, 134.8, 134.0, 129.8, 129.3, 128.9, 128.3, 126.4, 124.0, 123.8, 123.6(2), 67.2, 64.8, 53.2, 46.9, 40.2, 35.5, 29.4; HRMS (ESI+): m/z calcd. for C_57_H_54_Cl_2_N_12_O_4_^+^: 1041.3840, found: 1041.3810.

*6,6′-((Propane-1,3-diylbis(1H-1,2,3-triazole-1,4-diyl))bis(methylene))bis(2-benzyl-3-morpholino-7-propyl-6,7-dihydro-5H-pyrrolo[3,4-b]pyridin-5-one)* (**13d**): According to GP-2, **11d** (50 mg, 0.0011 μmol), 1,3-diazido propane (**12**) (8.1 mg, 0.059 μmol), CuSO_4_•5H_2_O (3.2 mg), and Na ascorbate (7.63 mg, 0.006 μmol) were reacted together in 1.0 mL DMF:H_2_O (1:1) in MW to afford the propane-linked bis-triazolyl-pyrrolo[3,4-b]pyridin-5-ones (**13d**) (83 mg, 71%) as yellow solid; m.p. 178–179 °C; DCM-MeOH = 9/1 v/v; ^1^H-NMR (500 MHz, CDCl_3_) δ 7.73 (s, 2H), 7.62 (s, 2H), 7.20–7.14 (m, 8H), 7.10–7.07 (m, 2H), 5.11 (d, J = 15.4 Hz, 2H), 4.58–4.51 (m, 2H), 4.44 (d, J = 15.4 Hz, 2H), 4.32–4.20 (m, 8H), 3.77–3.72 (m, 8H), 2.80–2.68 (m, 8H), 2.19–2.12 (m, 2H), 2.00–1.90 (m, 2H), 1.12–0.98 (m, 2H), 0.79–0.70 (m, 8H); ^13^C-NMR (126 MHz, CDCl_3_) δ 167.3, 161.7, 160.6, 147.6, 144.1, 139.6, 129.0, 128.4, 126.3, 124.5, 123.6, 67.3, 61.0, 53.2, 47.0, 40.1, 35.6, 31.5, 30.6, 16.2, 14.0; HRMS (ESI+): m/z calcd. for C_51_H_60_N_12_O_4_^+^: 905.4933, found: 905.4911.

*6,6′-((Propane-1,3-diylbis(1H-1,2,3-triazole-1,4-diyl))bis(methylene))bis(2-benzyl-7-(3,4-dimethoxyphenyl)-3-(piperidin-1-yl)-6,7-dihydro-5H-pyrrolo[3,4-b]pyridin-5-one)* (**13e**): According to GP-2, **11e** (50 mg, 0.0011 μmol), 1,3-diazido propane (**12**) (6.5 mg, 0.059 μmol), CuSO_4_•5H_2_O (2.5 mg), and Na ascorbate (6.17 mg, 0.003 μmol) were reacted together in 1.0 mL DMF:H_2_O (1:1) in MW to afford the propane-linked bis-triazolyl-pyrrolo[3,4-b]pyridin-5-ones (**13e**) (91 mg, 80%) as brown solid; m.p. 212–214 °C; DCM-MeOH = 9/1 v/v; ^1^H-NMR (400 MHz; CDCl_3_; 25^o^C; TMS): δ 7.80 (s, 2H), 7.60 (s, 2H), 7.24–7.19 (m, 4H, Ar-H), 7.17–7.09 (m, 6H, Ar-H), 6.91–6.83 (m, 4H, Ar-H), 6.63 (s, 2H), 5.53 (s, 2H), 5.17 (d, 2H, J = 15.4 Hz), 4.33–4.22 (m, 6H), 4.22–4.15 (m, 4H), 3.88 (s, 6H), 3.77 (s, 6H), 2.88–2.77 (m, 8H), 2.53–2.40 (m, 2H), 1.74–1.77 (m, 8H), 1.60–1.54 (m, 4H); ^13^C-NMR (126 MHz, CDCl_3_) δ 167.2, 162.3, 159.7, 149.4, 149.4, 149.3, 144.0, 139.6, 128.9, 128.0, 126.0, 123.6, 123.3, 123.1, 121.2, 111.4, 110.9, 65.1, 56.0, 55.9, 54.3, 46.8, 39.9, 35.2, 30.4, 26.4, 23.9; HRMS (ESI+): m/z calcd. for C_63_H_68_N_12_O_6_^+^: 1089.5457, found: 1089.5435.

*6,6′-((Propane-1,3-diylbis(1H-1,2,3-triazole-1,4-diyl))bis(methylene))bis(2-benzyl-7-(4-chlorophenyl)-3-(piperidin-1-yl)-6,7-dihydro-5H-pyrrolo[3,4-b]pyridin-5-one)* (**13f**): According to GP-2, **11f** (50 mg, 0.0010 μmol), 1,3-diazido propane (**12**) (6.9 mg, 0.054 μmol), CuSO_4_•5H_2_O (2.7 mg), and Na ascorbate (6.52 mg, 0.003 μmol) were reacted together in 1.0 mL DMF:H_2_O (1:1) in MW to afford the propane-linked bis-triazolyl-pyrrolo[3,4-b]pyridin-5-ones (**13f**) (78 mg, 69%) as yellow solid; m.p. 217–218 °C; DCM-MeOH = 9/1 v/v; ^1^H-NMR (500 MHz, CDCl_3_) δ 7.72 (s, 2H), 7.53 (s, 2H), 7.26 (d, J = 8.4 Hz, 2H), 7.14–7.01 (m, 14H), 5.49 (s, 2H), 5.11 (d, J = 15.4 Hz, 2H), 4.26–4.16 (m, 6H), 4.06 (d, J = 13.8 Hz, 4H), 2.75–2.64 (m, 8H), 2.45–2.35 (m, 2H), 1.69–1.56 (m, 8H), 1.53–1.46 (m, 4H); ^13^C-NMR (126 MHz, CDCl_3_) δ 167.5, 162.6, 159.3, 149.6, 143.8, 139.5, 134.7, 134.2, 129.9, 129.3, 129.0, 128.2, 126.2, 123.5, 123.3, 64.7, 54.4, 46.9, 39.9, 35.4, 30.5, 29.4, 26.5, 24.0; HRMS (ESI+): m/z calcd. for C_59_H_58_Cl_2_N_12_O_2_^+^: 1037.4255, found: 1037.4225.

*6,6′-((Propane-1,3-diylbis(1H-1,2,3-triazole-1,4-diyl))bis(methylene))bis(2-benzyl-3-(diethylamino)-7-phenyl-6,7-dihydro-5H-pyrrolo[3,4-b]pyridin-5-one)* (**13g**): According to GP-2, **11g** (50 mg, 0.0012 μmol), 1,3-diazido propane **12** (7.7 mg, 0.061 μmol), CuSO_4_•5H_2_O (3.2 mg), and Na ascorbate (7.2 mg, 0.003 μmol) were reacted together in 1.0 mL DMF:H_2_O (1:1) in MW to afford the propane-linked bis-triazolyl-pyrrolo[3,4-b]pyridin-5-ones (**13g**) (58 mg, 49%) as yellow solid; m.p. 187–189 °C; DCM-MeOH = 9/1 v/v; ^1^H-NMR (500 MHz, CDCl_3_) δ 7.82 (s, 2H), 7.59 (s, 2H), 7.39–7.33 (m, 6H), 7.26–7.22 (m, 4H), 7.16–7.08 (m, 10H), 5.59 (s, 2H), 5.20 (d, J = 15.4 Hz, 2H), 4.33–4.25 (m, 6H), 4.19–4.12 (m, 4H), 2.95 (q, J = 7.1 Hz, 8H), 2.53–2.42 (m, 2H), 0.89 (t, J = 7.1 Hz, 12H); ^13^C-NMR (126 MHz, CDCl_3_) δ 167.5, 163.8, 160.0, 146.5, 144.0, 139.6, 135.5, 129.1, 129.1, 128.8, 128.4, 128.1, 126.0, 125.7, 123.5, 123.4, 65.5, 47.9, 46.9, 40.0, 35.5, 30.6, 12.2; HRMS (ESI+): m/z calcd. for C_57_H_60_N_12_O_2_^+^: 945.5034, found: 945.5009.

*6,6′-((Propane-1,3-diylbis(1H-1,2,3-triazole-1,4-diyl))bis(methylene))bis(2-benzyl-3-(diethylamino)-7-(3,4-dimethoxyphenyl)-6,7-dihydro-5H-pyrrolo[3,4-b]pyridin-5-one)* (**13h**): According to GP-2, **11h** (50 mg, 0.0010 μmol), 1,3-diazido propane (**12**) (6.7 mg, 0.053 μmol), CuSO_4_•5H_2_O (3.2 mg), and Na ascorbate (6.33 mg, 0.0031 μmol) were reacted together in 1.0 mL DMF:H_2_O (1:1) in MW to afford the propane-linked bis-triazolyl-pyrrolo[3,4-b]pyridin-5-ones (**13h**) (61 mg, 53%) as orange solid; m.p. 191–194 °C; DCM-MeOH = 9/1 v/v; ^1^H-NMR (500 MHz, CDCl_3_) δ 7.82 (s, 2H), 7.62 (s, 2H), 7.18–7.06 (m, 10H), 6.93–6.83 (m, 4H), 6.63 (s, 2H), 5.55 (s, 2H), 5.18 (d, J = 15.4 Hz, 2H), 4.35–4.25 (m, 6H), 4.22–4.16 (m, 4H), 3.88 (s, 6H), 3.78 (s, 6H), 2.95 (q, J = 7.1 Hz, 8H), 2.53–2.41 (m, 2H), 0.89 (t, J = 7.1 Hz, 12H); ^13^C-NMR (126 MHz, CDCl_3_) δ 167.4, 163.9, 160.1, 149.5, 149.5, 146.5, 144.1, 139.7, 129.1, 128.1, 127.9, 127.7, 126.0, 125.7, 123.5, 123.4, 121.3, 111.4, 111.0, 65.3, 56.1 (2), 47.9, 46.9, 40.1, 35.4, 29.8, 12.2; HRMS (ESI+): m/z calcd. for C_61_H_68_N_12_O_6_^+^: 1065.5457, found: 1065.5424.

*6,6′-((Propane-1,3-diylbis(1H-1,2,3-triazole-1,4-diyl))bis(methylene))bis(2-benzyl-7-(4-chlorophenyl)-3-(diethylamino)-6,7-dihydro-5H-pyrrolo[3,4-b]pyridin-5-one)* (**13i**): According to GP-2, **11i** (46 mg, 0.0011 μmol), 1,3-diazido propane (**12**) (7.1 mg, 0.056 μmol), CuSO_4_•5H_2_O (2.8 mg), and Na ascorbate (6.69 mg, 0.003 μmol) were reacted together in 1.0 mL DMF:H_2_O (1:1) in MW to afford the propane-linked bis-triazolyl-pyrrolo[3,4-b]pyridin-5-ones (**13i**) (51 mg, 44%) as beige solid; m.p. 189–191 °C; DCM-MeOH = 9/1 v/v. ^1^H-NMR (500 MHz, CDCl_3_) δ 7.74 (s, 2H), 7.54 (s, 2H), 7.27 (d, J = 8.5 Hz, 4H), 7.12 (d, J = 8.6 Hz, 4H), 7.09–7.03 (m, 10H), 5.51 (s, 2H), 5.13 (d, J = 15.4 Hz, 2H), 4.26–4.18 (m, 6H), 4.11–4.05 (m, 4H), 2.88 (q, J = 7.1 Hz, 8H), 2.46–2.36 (m, 2H), 0.83 (t, J = 7.1 Hz, 12H); ^13^C-NMR (126 MHz, CDCl_3_) δ 167.4, 163.9, 159.4, 146.6, 143.6, 139.4, 134.6, 134.0, 129.7, 129.2, 129.0, 128.0, 126.0, 125.6, 123.1, 64.6, 47.7, 46.8, 39.8, 35.3, 29.3, 12.1; HRMS (ESI+): m/z calcd. for C_61_H_68_N_12_O_6_^+^: 1065.5457, found: 1065.5424.

*6,6′-((Propane-1,3-diylbis(1H-1,2,3-triazole-1,4-diyl))bis(methylene))bis(2-benzyl-3-(diethylamino)-7-propyl-6,7-dihydro-5H-pyrrolo[3,4-b]pyridin-5-one)* (**13j**): According to GP-2, **11j** (50 mg, 0.0013 μmol), 1,3-diazido propane (**12**) (8.4 mg, 0.066 μmol), CuSO_4_•5H_2_O (3.2 mg), and Na ascorbate (7.9 mg, 0.0039 μmol) were reacted together in 1.0 mL DMF:H_2_O (1:1) in MW to afford the propane-linked bis-triazolyl-pyrrolo[3,4-b]pyridin-5-ones (**13j**) (66 mg, 57%) as yellow solid; m.p. 173–174 °C; DCM-MeOH = 9/1 v/v; ^1^H-NMR (500 MHz, CDCl_3_) δ 7.71 (s, 2H), 7.62 (s, 2H), 7.20–7.05 (m, 10H), 5.11 (d, J = 15.4 Hz, 2H), 4.50 (dd, J = 5.9, 3.3 Hz, 2H), 4.44 (d, J = 15.4 Hz, 2H), 4.29 (d, J = 14.0 Hz, 1H), 4.27–4.22 (m, 4H), 4.19 (d, J = 14.0 Hz, 2H), 2.89 (q, J = 7.1 Hz, 8H), 2.47–2.38 (m, 2H), 1.98–1.88 (m, 4H), 1.13–0.98 (m, 4H), 0.84 (t, J = 7.1 Hz, 12H), 0.71 (t, J = 7.0 Hz, 6H); ^13^C-NMR (126 MHz, CDCl_3_) δ 167.5, 163.1, 159.9, 146.0, 144.0, 139.8, 129.0, 128.0, 125.9, 125.4, 123.8, 123.5, 60.9, 47.9, 46.9, 39.8, 35.5, 31.4, 30.5, 16.1, 13.9, 12.1; HRMS (ESI+): m/z calcd. for C_51_H_64_N_12_O_2_^+^: 877.5347, found: 877.5314.

## 4. Conclusions

The MCR-based domino processes coupled to other synthetic tools, such as a double-click reaction, is an excellent alternative for designing and developing novel, efficient, and more eco-friendly synthetic strategies. The novel strategy developed herein involves an MCR coupled to a domino followed by a tandem process toward the synthesis of polyheterocyclic dimers. In addition, to the best of our knowledge, this is the first report on the synthesis of 1,2,3-triazoles and pyrrolo[3,4-*b*]pyridine dimers linked to PHPs via a MCR-based domino followed by the double CuAAC click sequence. The integration of three highly efficient, convergent, versatile, and robust synthetic tools made the developed strategy one of the best alternatives toward the synthesis of polyheterocyclic dimers, which are of particular interest in biological and medicinal chemistry as they contain privileged heterocyclic restricted and constrained peptidomimetics. Interestingly, the developed strategy efficiently allowed the construction of six heterocycles in only two experimental steps. In the same way, the methodology reported here contributes majorly to the field of designing and synthesis novel molecules with potential application in biological, medicinal chemistry, and optics.

## Figures and Tables

**Table 1:** Substrate scope.

Entry	R^1^	R^2^	11 (%) *^a^*	13 (%) *^a^*
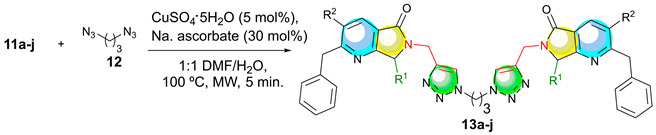				
1	Ph	morpholine	**11a**, 64	**13a**, 50
2	3,4-MeOPh	morpholine	**11b**, 62	**13b**, 63
3	4-Cl-Ph	morpholine	**11c**, 53	**13c**, 79
4	*n*-propyl	morpholine	**11d**, 47	**13d**, 71
5	3,4-MeOPh	piperidine	**11e**, 66	**13e**, 80
6	4-Cl-Ph	piperidine	**11f**, 56	**13f**, 69
7	Ph	diethylamine	**11g**, 59	**13g**, 49
8	3,4-MeOPh	diethylamine	**11h**, 63	**13h**, 53
9	4-Cl-Ph	diethylamine	**11i**, 46	**13i**, 44
10	*n*-propyl	diethylamine	**11j**, 50	**13j**, 57

*^a^* Isolated product. DMF = *N,N*-Dimethylformamide.

## Supplementary Materials

The following are available online at, NMR-spectras and mass spectrometric data of the new products **13a**–**j** can be found in the Supporting Information.
