# Supplementary material for: Synthesis of Polyheterocyclic Dimers Containing Restricted and Constrained Peptidomimetics via IMCR-Based Domino/Double CuAAC Click Strategy
Source: Molecules. 2020 Nov 11;25(22):5246. doi: 10.3390/molecules25225246 (PMC7696539; doi:10.3390/molecules25225246)
Supplement: Supplementary file 1 [file molecules-25-05246-s001.pdf]

S U P P O R T I N G   I N F O R M A T I O N

"Synthesis of polyheterocyclic dimers containing restricted and constrained peptidomimetics via : IMCR-based domino/ double CuAAC click strategy"

Shrikant G. Pharande [1], Manuel A. Rentería-Gómez [1], Rocío Gámez-Montaño [1]*

[1]Departamento de Química, Universidad de Guanajuato, Guanajuato, Mexico

* rociogm@ugto.mx (R.G.-M.)

Characterization of the 6-propargyl-pyrrolo[3,4-*b*]pyridin-5-ones 11a-j:
For the characterizeation see: R. Gámez-Montaño*, *Front. Chem.* **2019**, 7:546.

Characterization of the propane-linked *bis*-triazolyl-pyrrolo[3,4-*b*]pyridin-5-ones 13a-j:

6,6'-((propane-1,3-diylbis(1*H*-1,2,3-triazole-1,4-diyl))bis(methylene))bis(2-benzyl-3-morpholino-7-phenyl-6,7-dihydro-5*H*-pyrrolo[3,4-*b*]pyridin-5-one) (**13a**)

^{1}H NMR **13a**

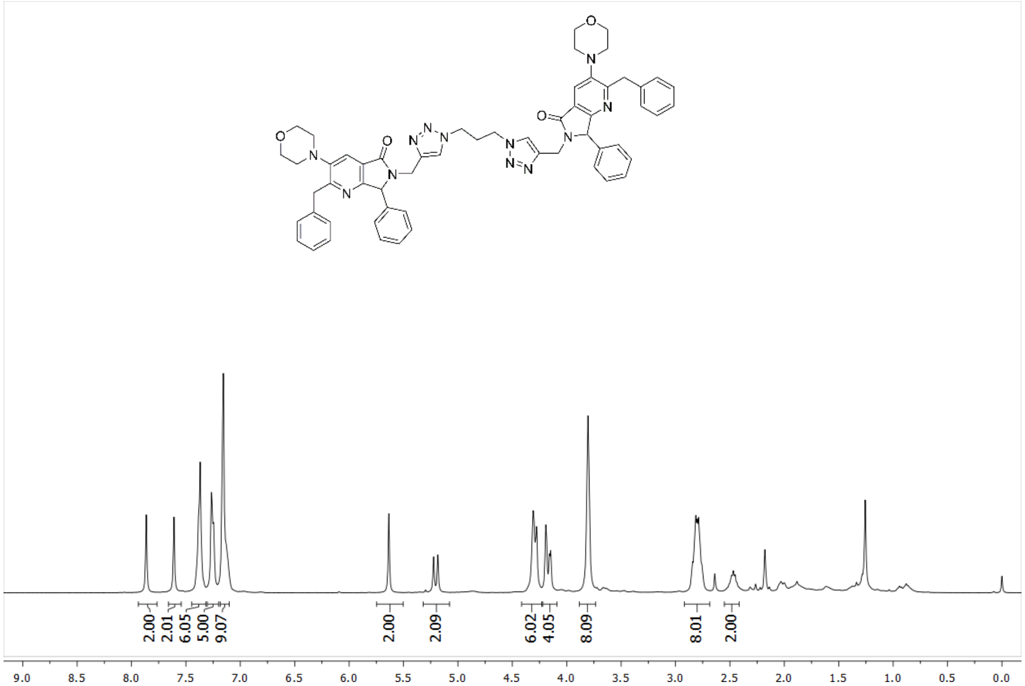

^{13}C NMR **13a**

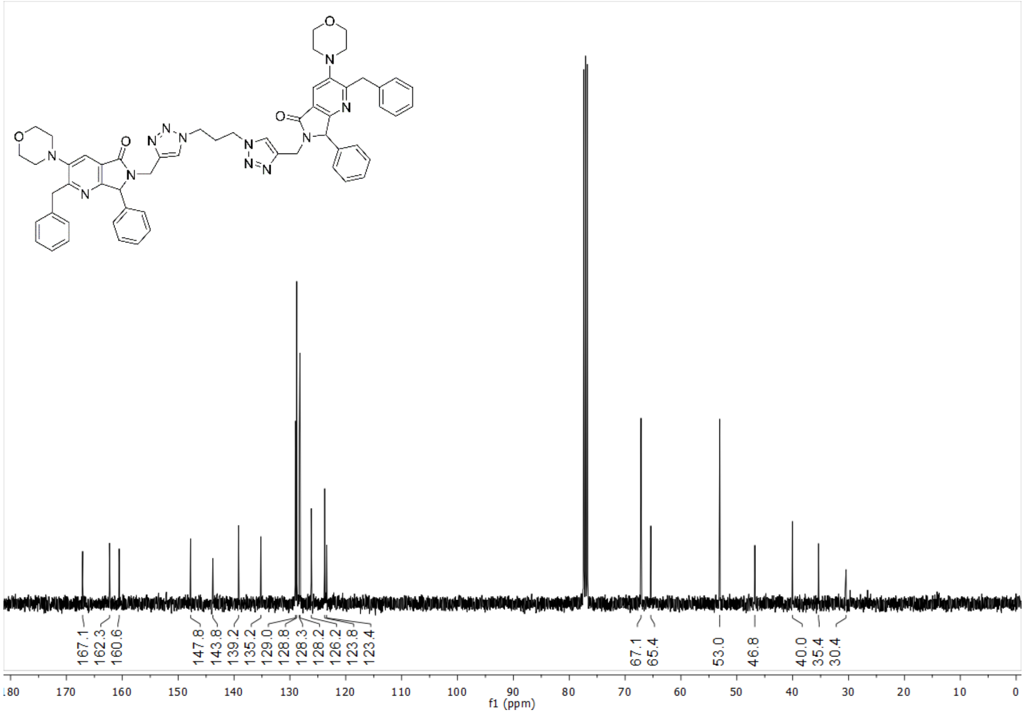

HRMS **13a**

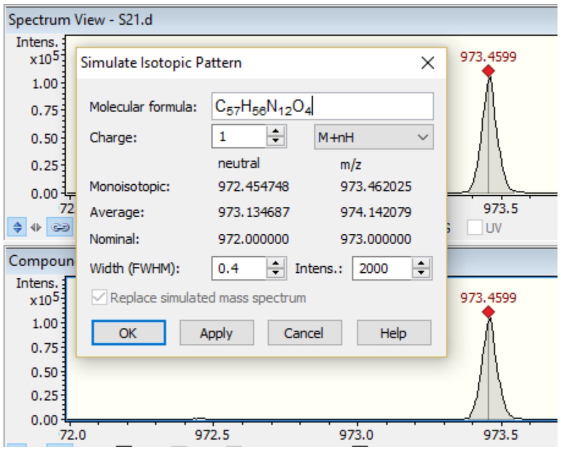

6,6'-((propane-1,3-diylbis(1*H*-1,2,3-triazole-1,4-diyl))bis(methylene))bis(2-benzyl-7-(3,4-dimethoxyphenyl)-3-morpholino-6,7-dihydro-5*H*-pyrrolo[3,4-*b*]pyridin-5-one) (**13b**)

^{1}H NMR **13b**

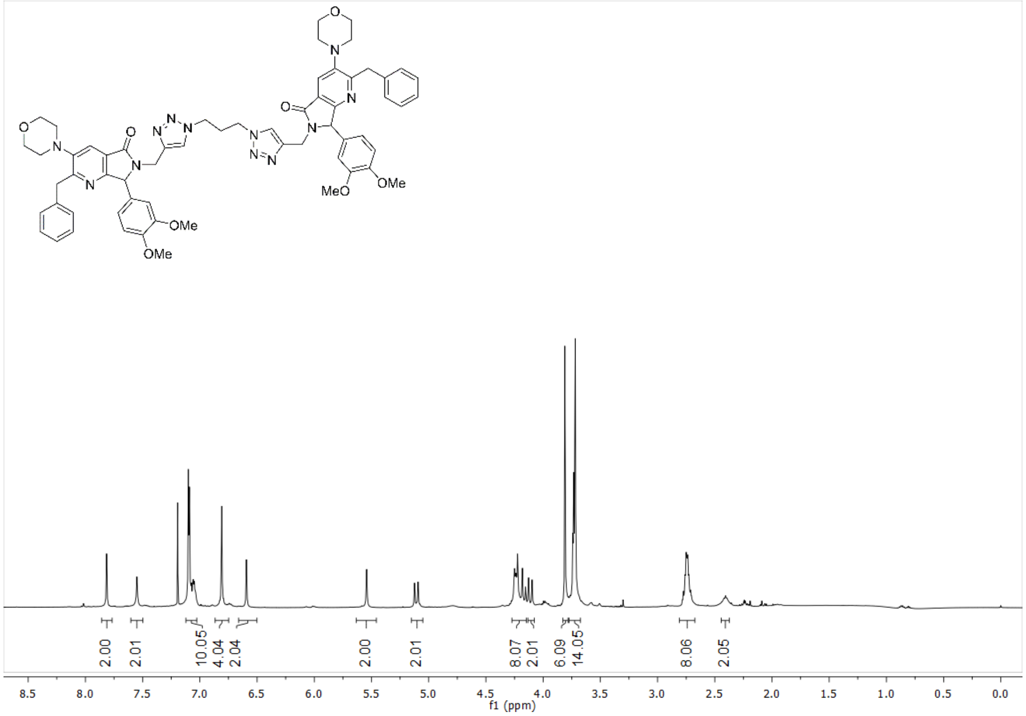

^{13}C NMR **13b**

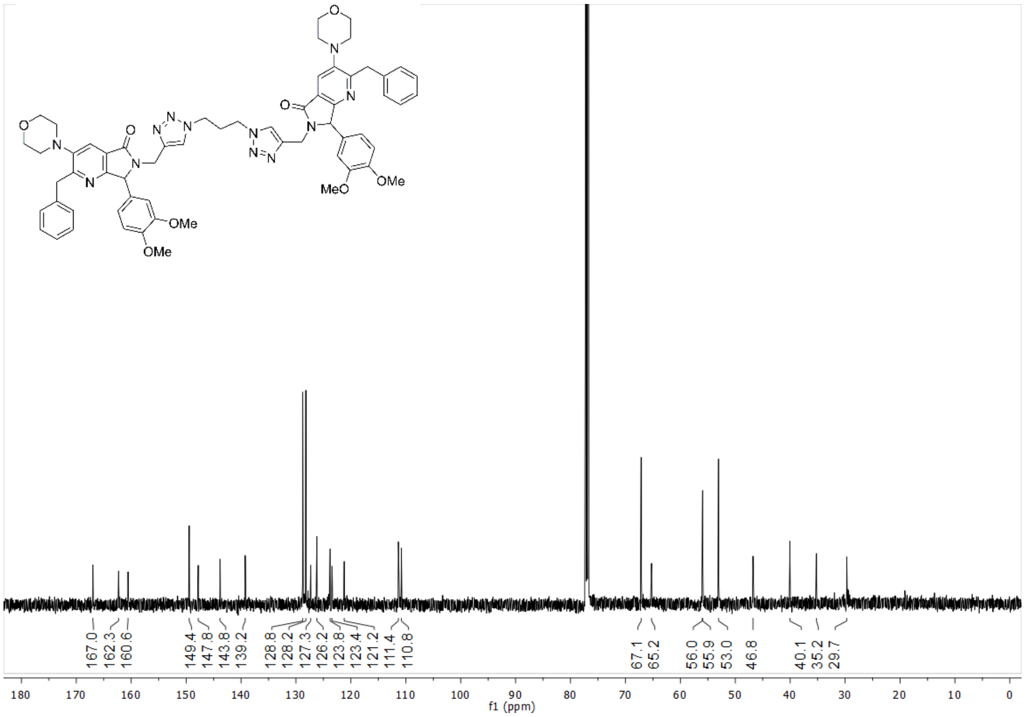

HRMS **13b**

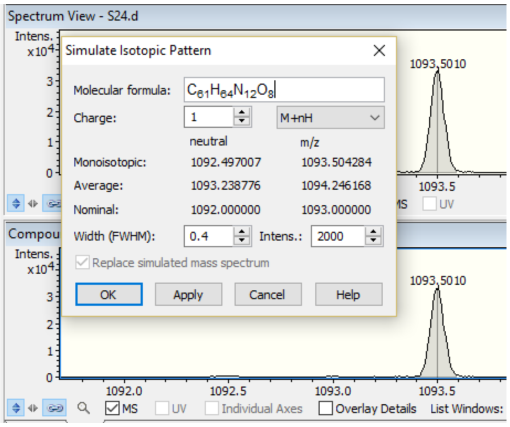

6,6'-((propane-1,3-diylbis(1*H*-1,2,3-triazole-1,4-diyl))bis(methylene))bis(2-benzyl-7-(4-chlorophenyl)-3-morpholino-6,7-dihydro-5*H*-pyrrolo[3,4-*b*]pyridin-5-one) (**13c**)

^{1}H NMR **13c**

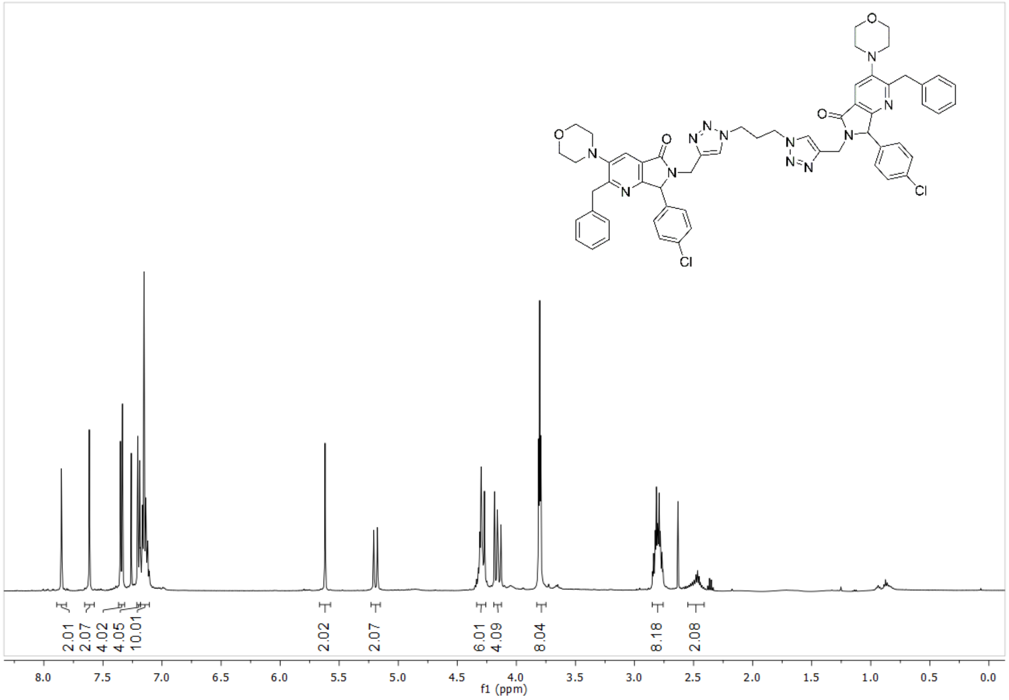

^{13}C NMR **13c**

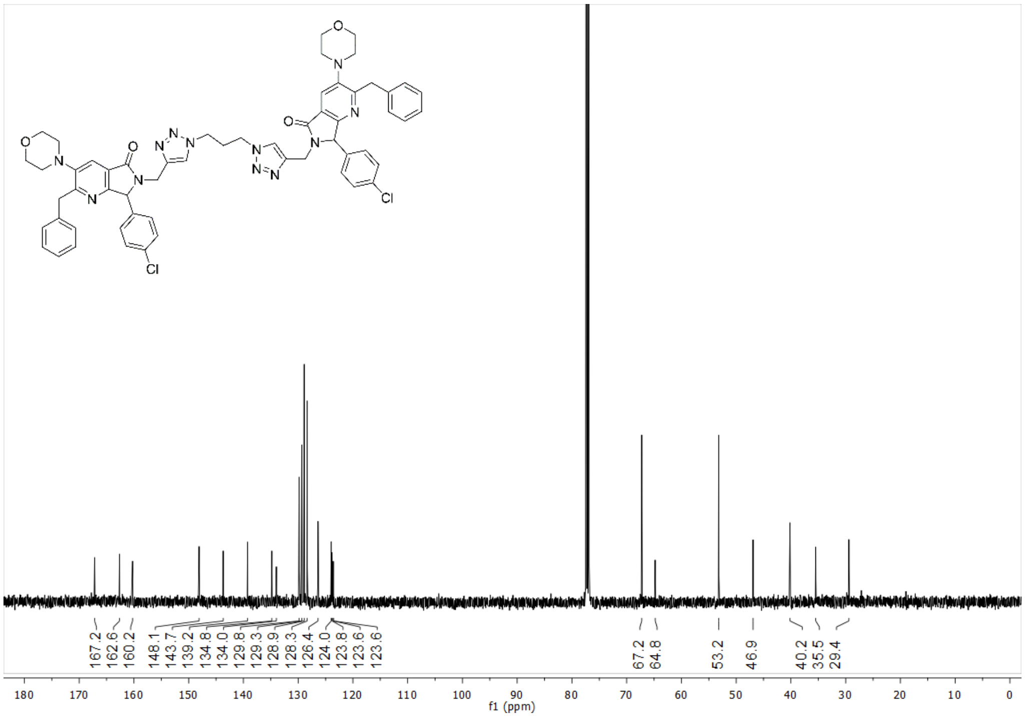

HRMS **13c**

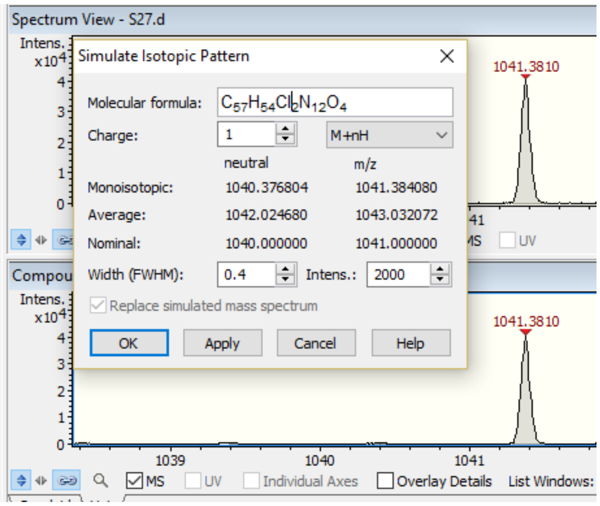

6,6'-((propane-1,3-diylbis(1*H*-1,2,3-triazole-1,4-diyl))bis(methylene))bis(2-benzyl-3-morpholino-7-propyl-6,7-dihydro-5*H*-pyrrolo[3,4-*b*]pyridin-5-one) (**13d**)

^{1}H NMR **13d**

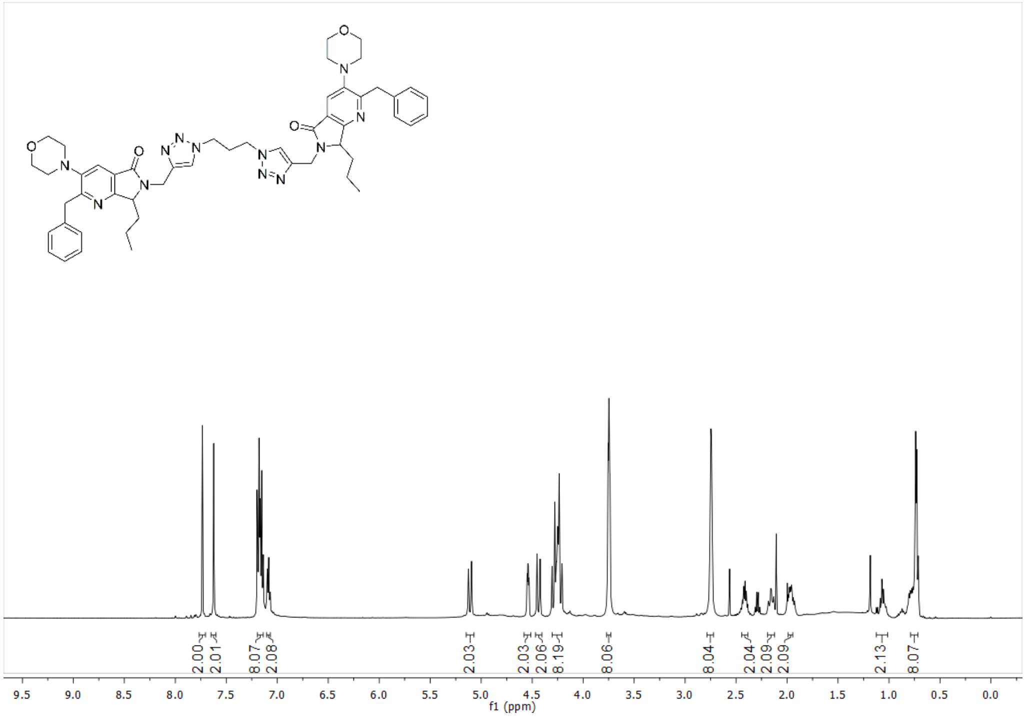

^{13}C NMR **13d**

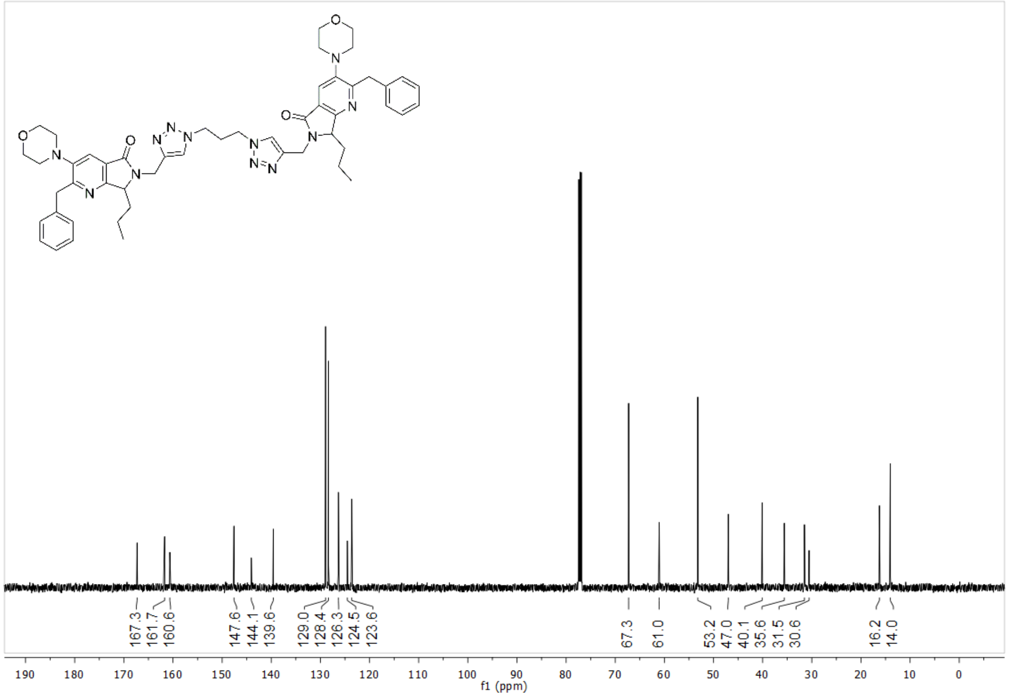

HRMS **13d**

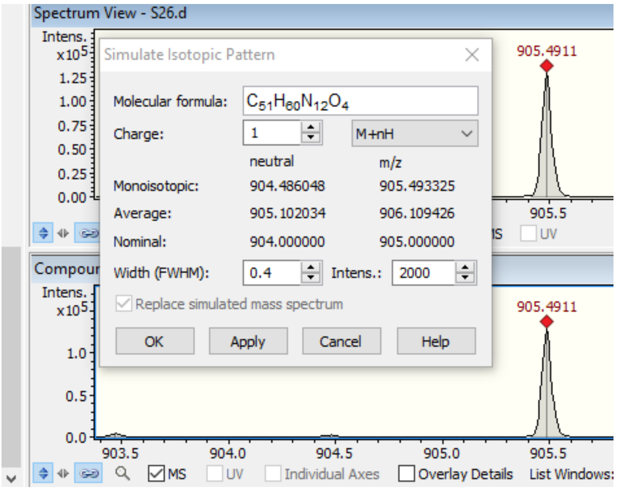

6,6'-((propane-1,3-diylbis(1*H*-1,2,3-triazole-1,4-diyl))bis(methylene))bis(2-benzyl-7-(3,4-dimethoxyphenyl)-3-(piperidin-1-yl)-6,7-dihydro-5*H*-pyrrolo[3,4-*b*]pyridin-5-one) (**13e**)

^{1}H NMR **13e**

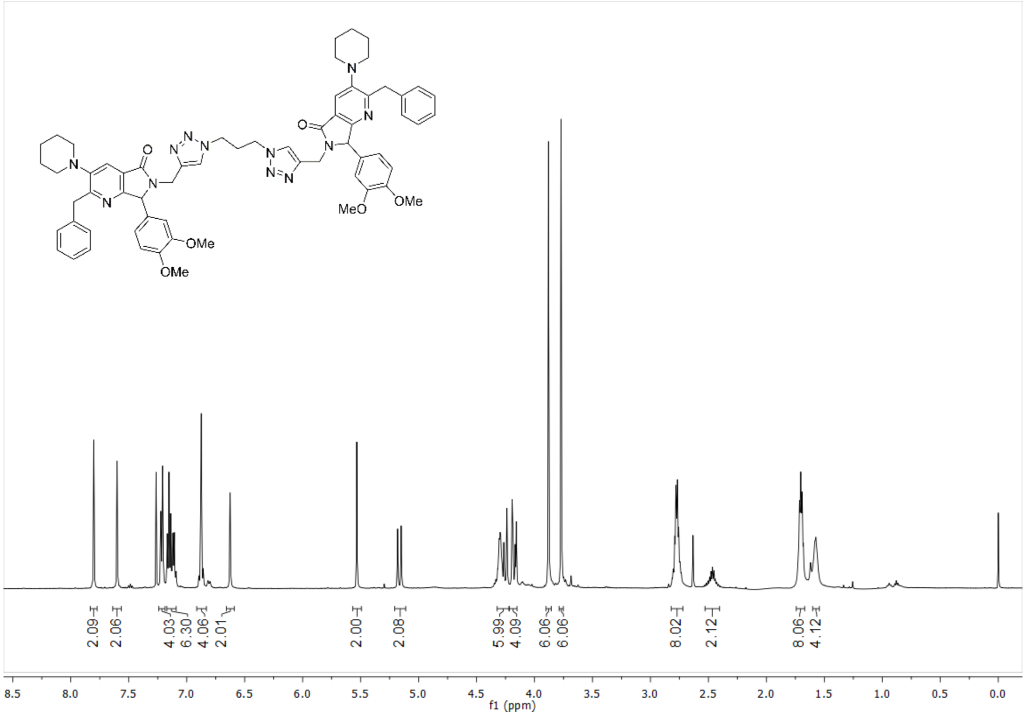

^{13}C NMR **13e**

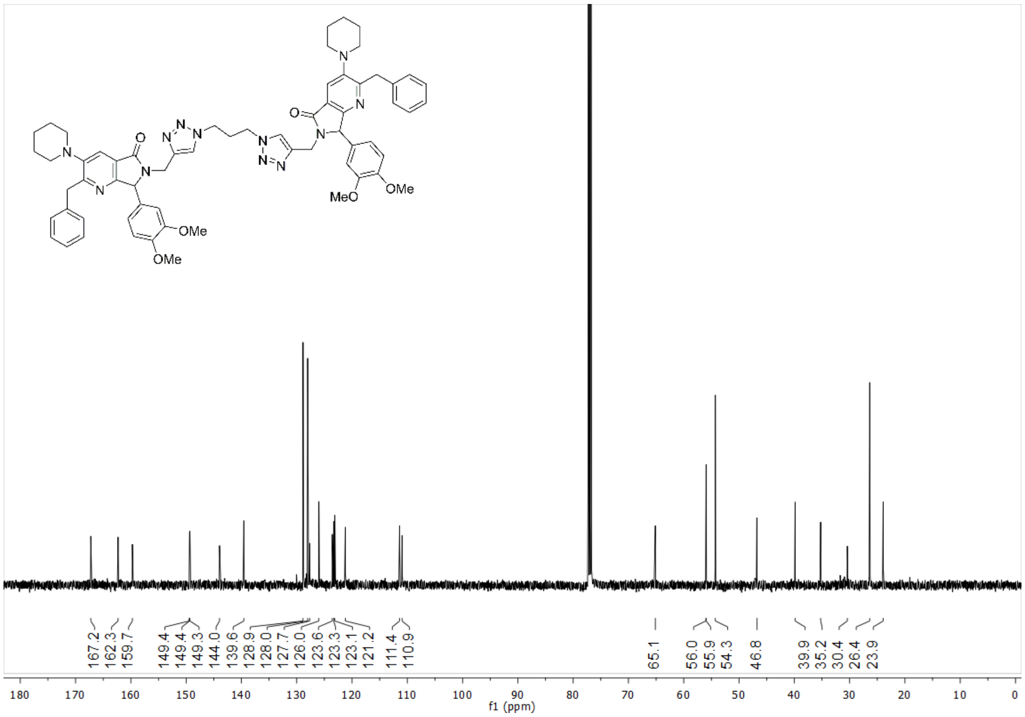

HRMS **13e**

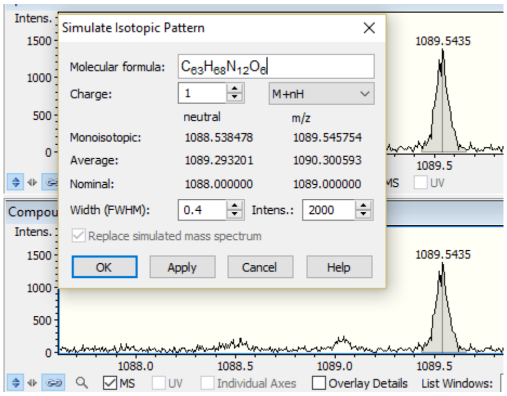

6,6'-((propane-1,3-diylbis(*1H*-1,2,3-triazole-1,4-diyl))bis(methylene))bis(2-benzyl-7-(4-chlorophenyl)-3-(piperidin-1-yl)-6,7-dihydro-5*H*-pyrrolo[3,4-*b*]pyridin-5-one) (**13f**)

^{1}H NMR **13f**

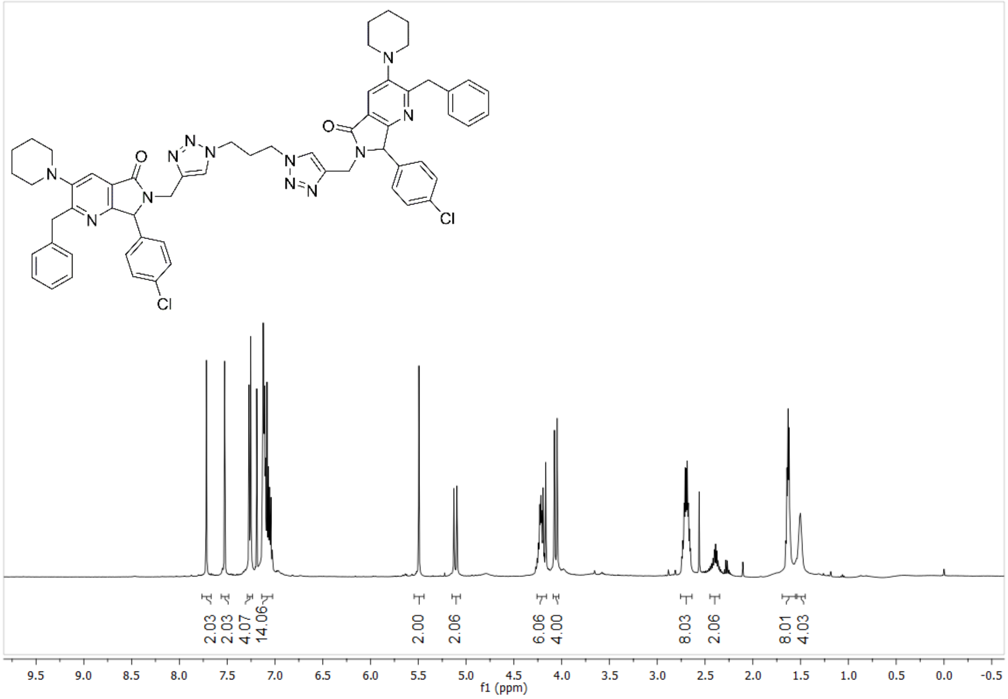

^{13}C NMR **13f**

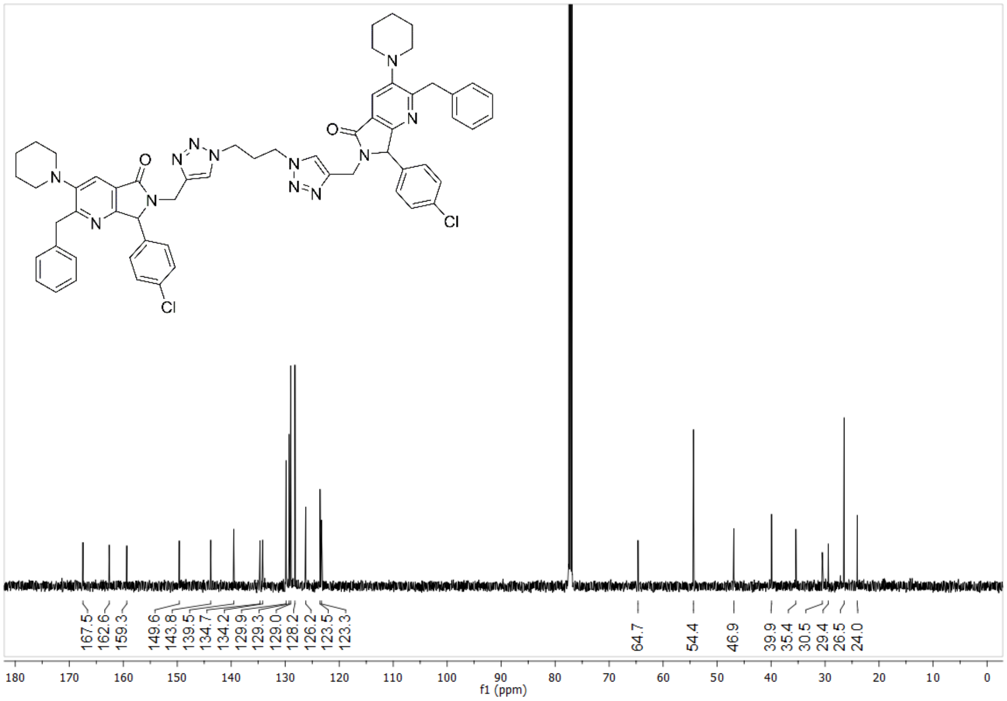

HRMS **13f**

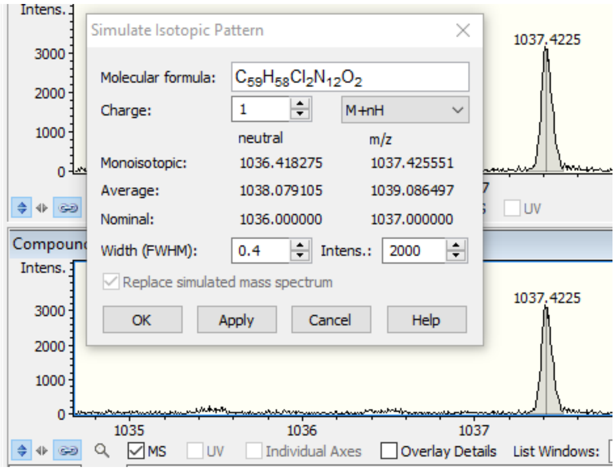

6,6'-((propane-1,3-diylbis(1*H*-1,2,3-triazole-1,4-diyl))bis(methylene))bis(2-benzyl-3-(diethylamino)-7-phenyl-6,7-dihydro-5*H*-pyrrolo[3,4-*b*]pyridin-5-one) (**13g**)

^{1}H NMR **13g**

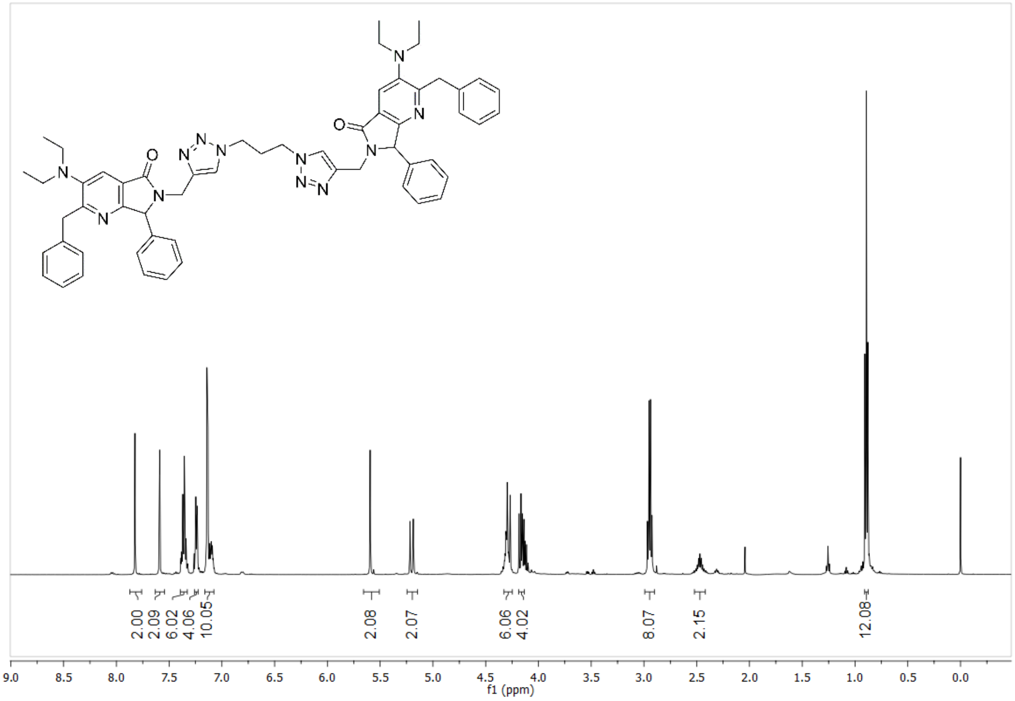

^{13}C NMR **13g**

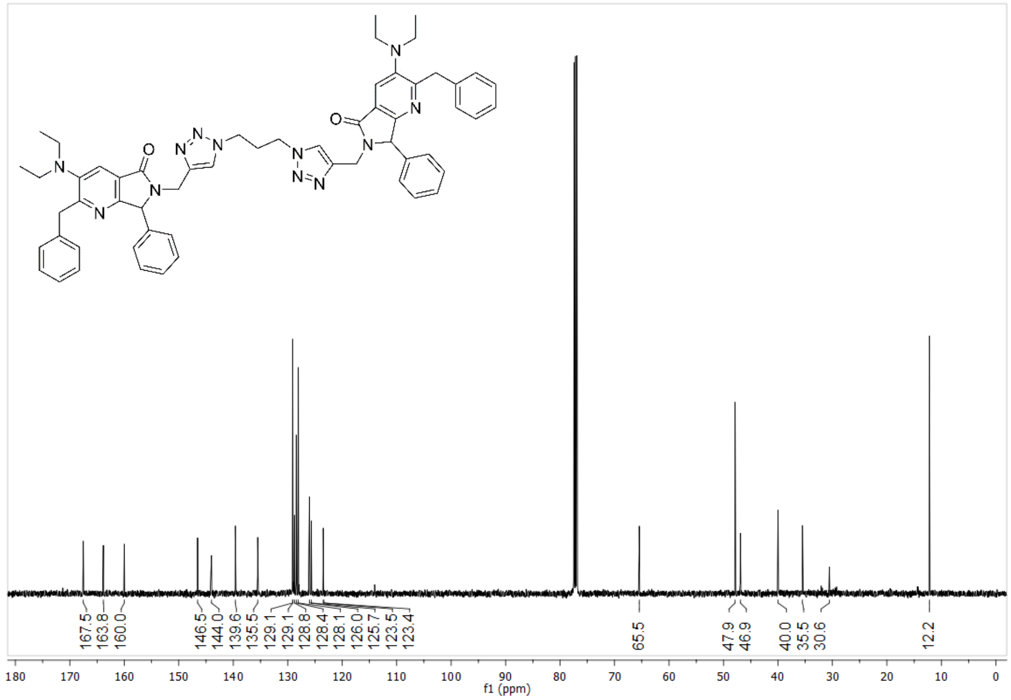

HRMS **13g**

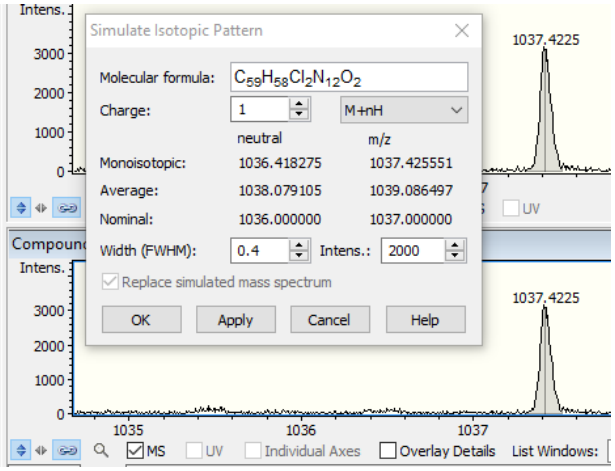

6,6'-((propane-1,3-diylbis(1H-1,2,3-triazole-1,4-diyl))bis(methylene))bis(2-benzyl-3-(diethylamino)-7-(3,4-dimethoxyphenyl)-6,7-dihydro-5*H*-pyrrolo[3,4-*b*]pyridin-5-one) 13h

^{1}H NMR **13h**

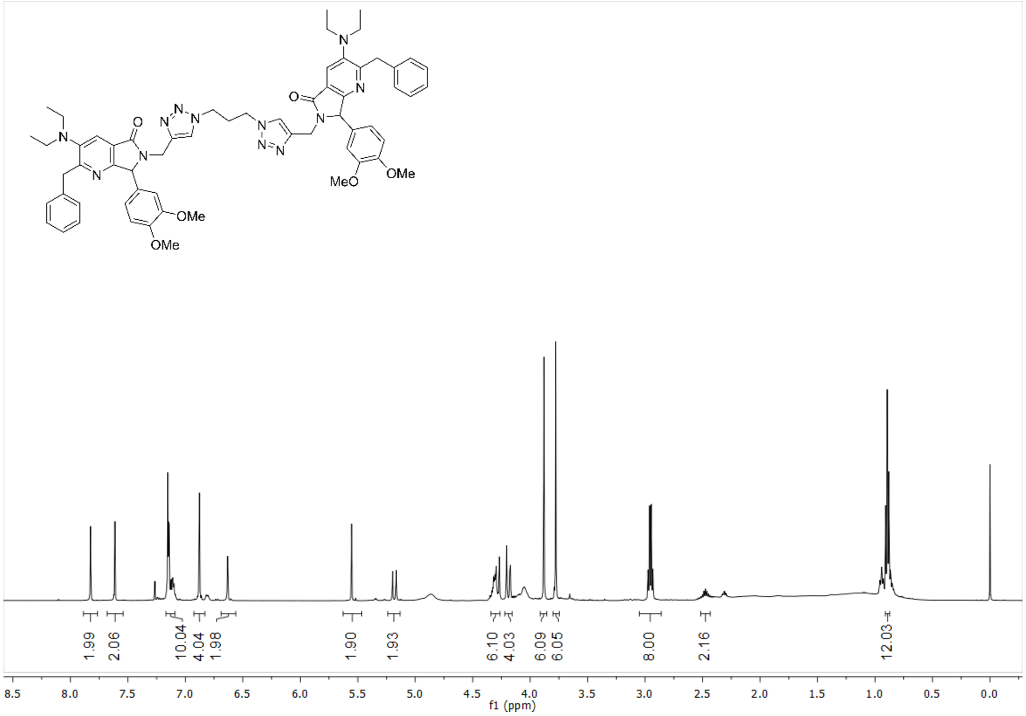

^{13}C NMR **13h**

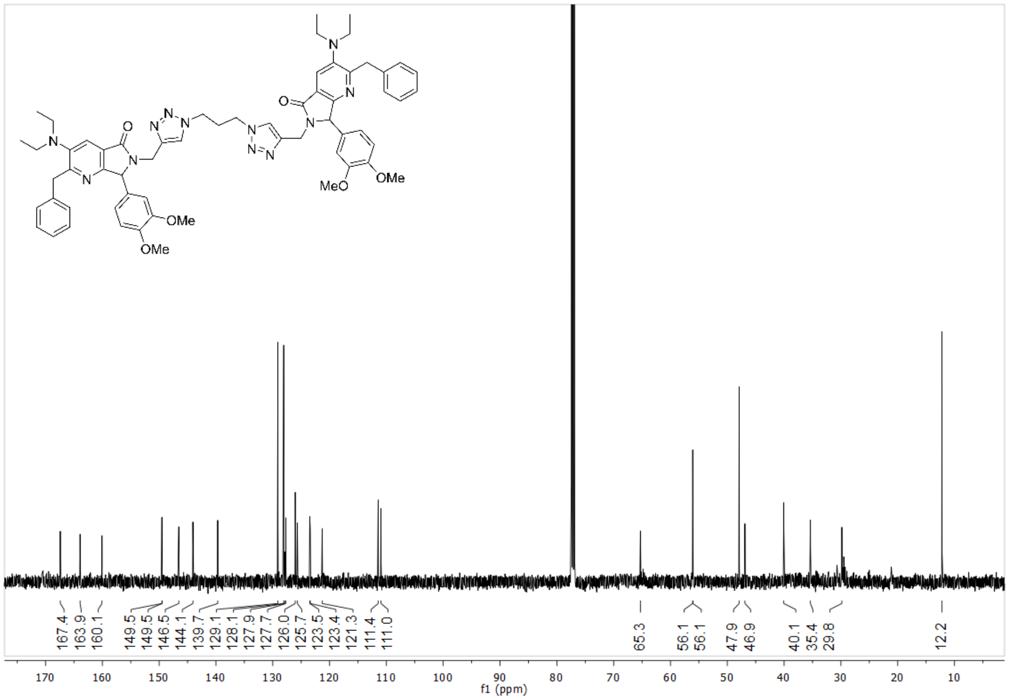

HRMS **13h**

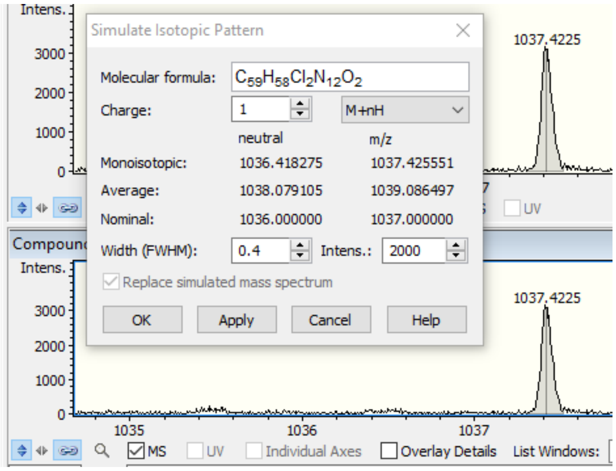

6,6'-((propane-1,3-diylbis(1H-1,2,3-triazole-1,4-diyl))bis(methylene))bis(2-benzyl-7-(4-chlorophenyl)-3-(diethylamino)-6,7-dihydro-5*H*-pyrrolo[3,4-*b*]pyridin-5-one) 13i

^{1}H NMR **13i**

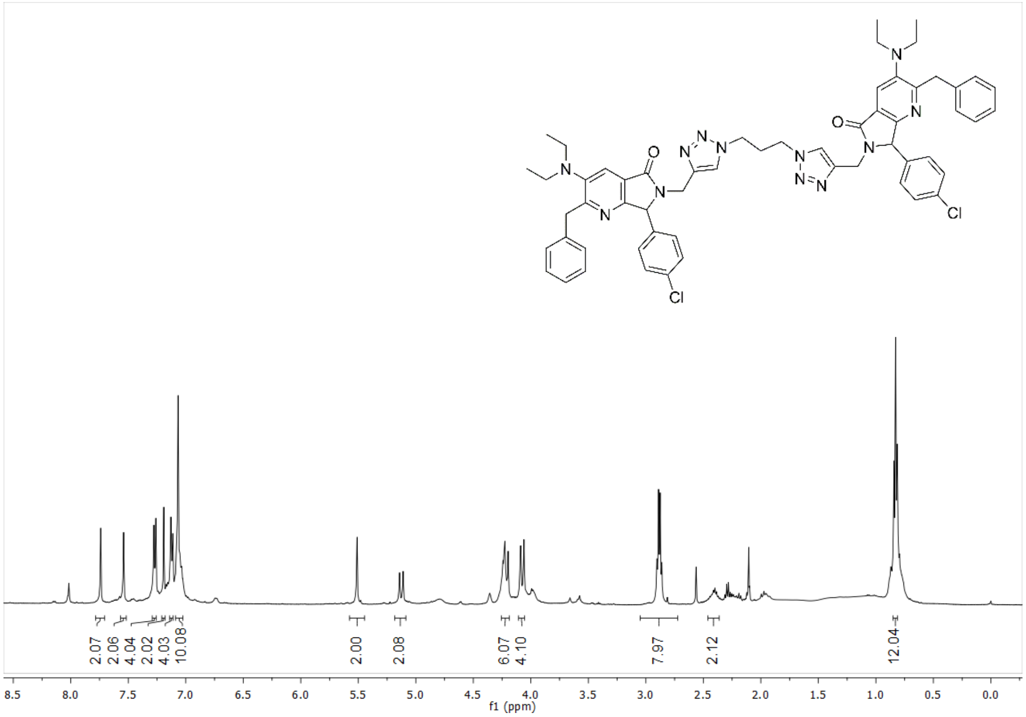

^{13}C NMR **13i**

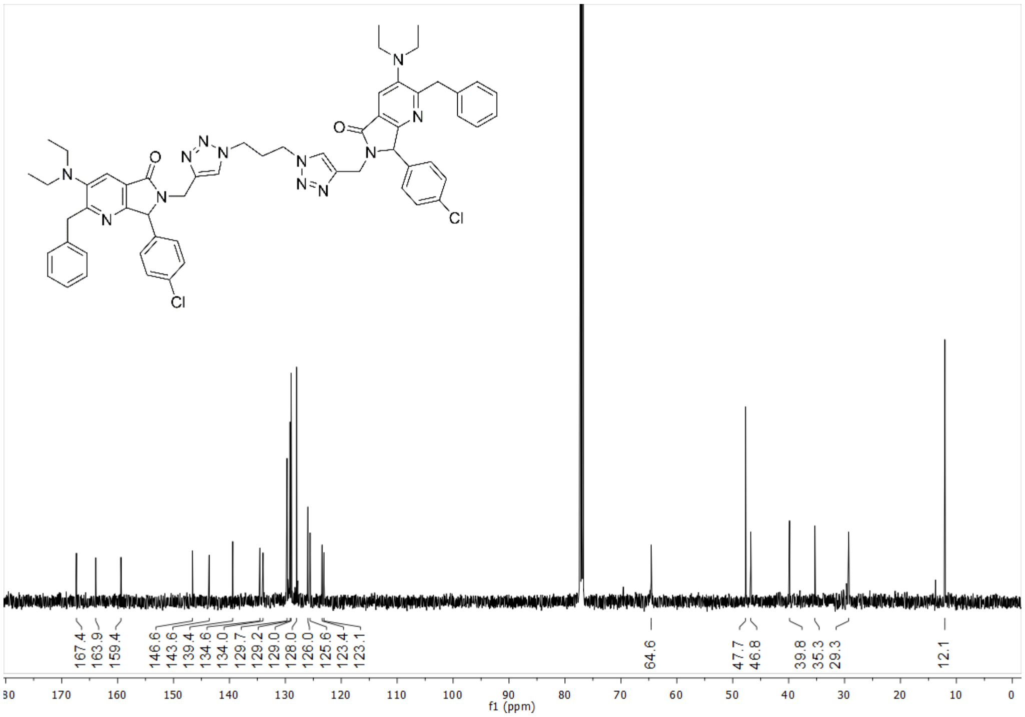

HRMS **13i**

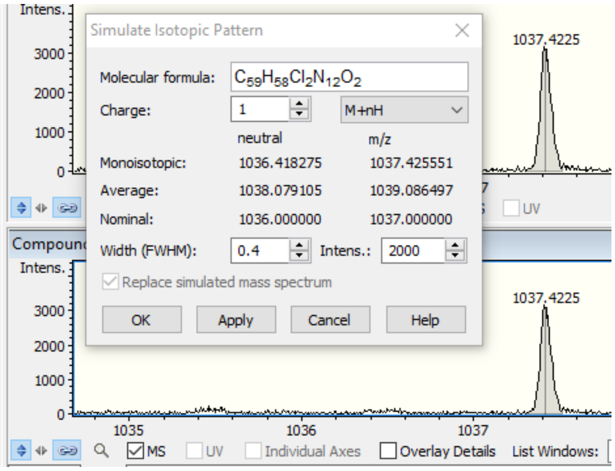

6,6'-((propane-1,3-diylbis(1H-1,2,3-triazole-1,4-diyl))bis(methylene))bis(2-benzyl-3-(diethylamino)-7-propyl-6,7-dihydro-5*H*-pyrrolo[3,4-*b*]pyridin-5-one) 13j

^{1}H NMR **13j**

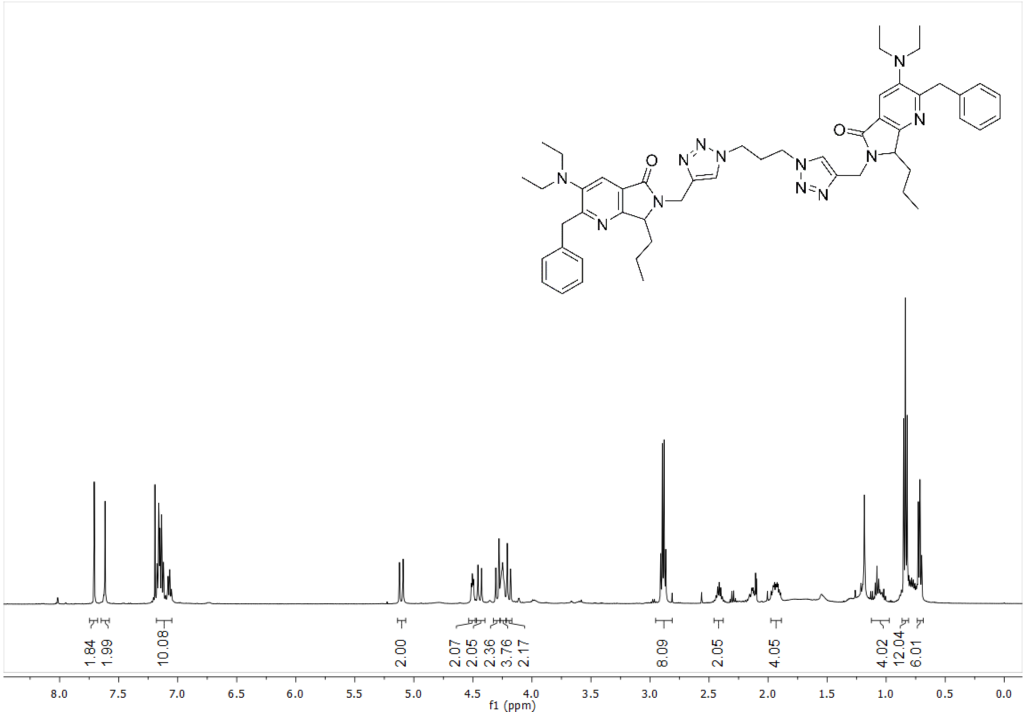

^{13}C NMR **13j**

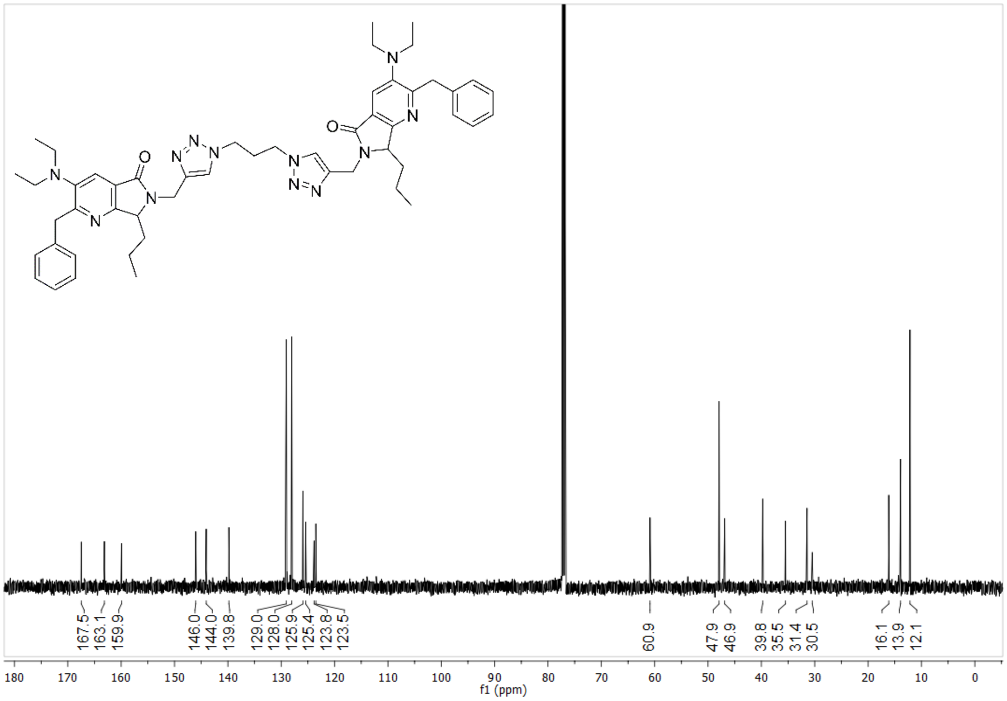

HRMS **13j**

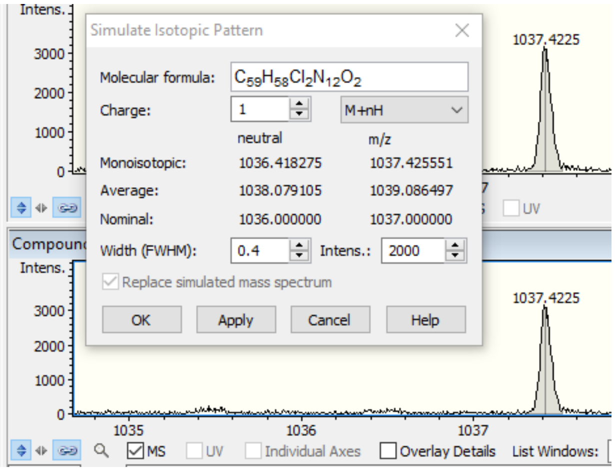
Simulate Isotopic Pattern
Molecular formula: C59H58Cl2N12O2
Charge: 1
M+nH
neutral
m/z
Monoisotopic: 1036.418275 1037.425551
Average: 1038.079105 1039.086497
Nominal: 1036.000000 1037.000000
Width (FWHM): 0.4
Intens.: 2000
Replace simulated mass spectrum
OK
Apply
Cancel
Help
1037.4225
1037.4225
Intens.
3000
2000
1000
0
1035
1036
1037
MS
UV
Individual Axes
Overlay Details
List Windows:
